# Comparison of percutaneous transforaminal endoscopic discectomy and open lumbar discectomy for lumbar disc herniations: A systematic review and meta-analysis

**DOI:** 10.3389/fsurg.2022.984868

**Published:** 2022-11-11

**Authors:** Jian Zhang, Yangyang Gao, Bin Zhao, Haoyang Li, Xuening Hou, Liqiang Yin

**Affiliations:** Department of Orthopaedic Surgery, Jincheng People’s Hospital, Jincheng, China

**Keywords:** lumbar disc herniation, open lumbar discectomy, percutaneous transforaminal endoscopic discectomy, meta-analysis, treatment outcome

## Abstract

**Purpose:**

In order to compare the outcomes of percutaneous transforaminal endoscopic discectomy (PTED) and open lumbar discectomy (OLD) for lumbar disc herniation (LDH).

**Methods:**

The Pubmed, Cochrane Library, Web of Sience, Embase, Clinicaltrials.gov, CBM, CNKI, VIP, Wangfang databases were searched from inception to April 30, 2022 to collect the published studies about PTED vs. OLD for treatment of LDH. The Revman 5.2 was used for data analysis. The primary outcomes were excellent rates, complication rates and reoperation rates. The secondary outcomes were length of incision, length of operation, length of hospital stay, and the amount of intraoperative blood loss.

**Results:**

A total of nine studies were included, of which, eight randomized controlled trials and one retrospective study involving 1,679 patients with LDH (755 patients for PTED, and 924 patients for OLD) were included. According to meta-analysis, there were no significant difference in excellent rates (odds ratio [OR] = 1.47, 95% confidence intervals [CI]: 0.94–2.28, *P* = 0.09), reoperation rates (OR = 0.96, 95% CI: 0.50–1.84, *P* = 0.90), length of operation [standardized mean differences (SMD) = −17.97, 95%CI: −54.83–18.89, *P* = 0.34], and the amount of intraoperative blood loss (SMD = −128.05, 95%CI: −258.67–2.57, *P* = 0.05), respectively. There were significant differences in complication rates (OR = 0.22, 95% CI: 0.14–0.33, *P* < 0.001), length of incision (SMD = −2.76, 95%CI: −2.88–−2.65, *P* < 0.001), and length of hospital stay (SMD = −5.19, 95%CI: −5.36–−5.01, *P* < 0.001), respectively.

**Conclusions:**

PTED can achieve better outcomes with respect to the complication rates, length of incision, and length of hospital stay compared with OLD.

## Introduction

The prevalence of sciatica or radiculitis ranging from 1.2% to 43% worldwide (1). Sciatica is commonly caused by lumbar disc herniation (LDH), and the typical clinical presentation is pain radiating from the waist to the lower extremities, often accompanied by sensory or motor disturbances (2, 3). Most patients with sciatica have a good prognosis with conservative treatment. However, when conservative treatment fails, surgery may be required to relieve symptoms (4). In 1934, the first case of spinal surgery for the treatment of lumbar disc herniation was reported using open lumbar discectomy (OLD) (5). With the development of spine surgery techniques, spine surgeons are increasingly pursuing the treatment of LDH with less trauma (6). Therefore, traditional open minimally invasive surgery has gradually become the standard for the treatment of LDH (5, 7). With the development of endoscopic technology, its application in spine surgery is more and more extensive. Especially after Kambin et al. proposed the spinal safety zone, its combination with the endoscopic technique made various spinal minimally invasive techniques emerge as the times require (8, 9). Percutaneous transforaminal endoscopic discectomy (PTED) is one of spinal minimally invasive surgeries. Compared with traditional incision, PTED does not require dissection of paravertebral muscles, preserves the original bone anatomy, and can be performed with local anesthesia. It has a shorter hospital stay, less trauma, faster postoperative recovery, and better relief of pain, sensory, and motor symptoms (10). However, some scholars believe that PTED is not significantly different from traditional incisional discectomy (11–15). Therefore, the conclusions remain inconsistent. Previously, some meta-analyses comparing the effectiveness of endoscopic discectomy with OLD for LDH was performed. However, in those studies, some kinds of discectomy such as micro-endoscopic discectomy, PTED, and full-endoscopic discectomy together into endoscopic discectomy group and compared them with OLD surgery, which was actually not provided robust evidence (16, 17).

Herein, we aimed to conduct a comprehensively and systematically systematic literature review and meta-analysis to estimate pooled effect sizes, and compare the efficacy of PTED vs. OLD approach in the treatment of LDH.

## Methods

### Study selection

A systematic review of the English literature available on Pubmed, Cochrane Library, Web of Sience, Embase, and Clinicaltrials.gov was performed, along with a review of Chinese literature available on Chinese Biomedical database (CBM), Chinese National Knowledge Infrastructure (CNKI), Chinese Science and Technology Periodical database (VIP) and WanFang databases from inception to April 30, 2022. The query utilized in the search was designed to include as many literatures as possible pertaining to the outcomes of interest. The final search string was: “percutaneous transforaminal endoscopic discectomy” OR “open lumbar discectomy” OR “traditional discectomy” AND “lumbar disc herniation”. Articles which investigated operative approaches on LDH were identified without language restrictions. This study was performed according to the version of the Preferred Reporting Items for Systematic Reviews and Meta-Analyses (PRISMA) Statement (www.prisma-statement.org). The PRISMA Checklist was shown in Additional File 1.

### Inclusion and exclusion criteria

Articles were included according to the following criteria: (1) performed the comparison between PTED and OLD; (2) participants were adults who suffer LDH; (3) contained at least one outcome of interest; (4) patients without recurrent reoperation. Articles were excluded if: Interventions were different from the previous description; or insufficient data such as any outcome of interest; or not human studies. Additionally, patients with multi-segmental lumbar disc herniation, cauda equina syndrome, malignancy, or spinal deformity were also excluded. PTED introduced in 2002, is more minimally invasive, with posterior column lumbar structures preserved.

The primary outcomes were excellent rates, complication rates and reoperation rates. The secondary outcomes were length of incision, length of operation, length of hospital stay, and the amount of intraoperative blood loss.

### Literature screening

The literature obtained after searching the database was imported into the EndNote X9 literature management software, and the “Find Duplication” function of the software was used to remove the duplicated literature. Two researchers independently screened the titles and abstracts of the literature one by one according to the inclusion and exclusion criteria, and excluded those that did not meet the criteria. For the literatures with the inclusion criteria, the full text of the initially included literatures and the literatures that could not be determined to meet the inclusion criteria were reviewed. In case of disagreements, they were discussed and resolved to determine the final included literatures.

### Data extraction

Two researchers independently extracted data, and entered statistical software for statistical analysis. The data extraction includes characteristics of included studies: first author, publication year, study design, number of cases, age, and outcomes. The Jadad scoring tool was employed to assess the quality of evidence (18, 19). A Jadad scale score of 4–7 was classified as high-quality literature, and 1–3 was classified as low-quality literature. If there is a disagreement, a decision will be made through mutual consultation.

### Statistical analysis

The acquired data were analyzed using RevMan 5.2 software (The Nordic Cochrane Center, The Cochrane Collaboration, Denmark). The continuous outcomes were analyzed using standardized mean difference (SMD) and 95% confidence interval (CI). Odds ratio (OR) and 95% CI were used for dichotomous outcomes. *P*-value less than 0.05 was considered to be statistically significant. The heterogeneity analysis of the included literature was evaluated using Q-test (*χ*^2^) and *I*^2^. If the *P*-value was >0.05, and *I*^2^ < 50%, it was considered that there was no significant statistical heterogeneity among different studies, and a fixed effect model was used. If the *P* value <0.05 and *I*^2^ > 50%, statistical heterogeneity exists, and a random-effects model was used. In case of large heterogeneity, sensitivity analysis or subgroup analysis was performed. A funnel chart was used to evaluate possible publication bias qualitatively.

## Results

### Search results

**Figure 1:**
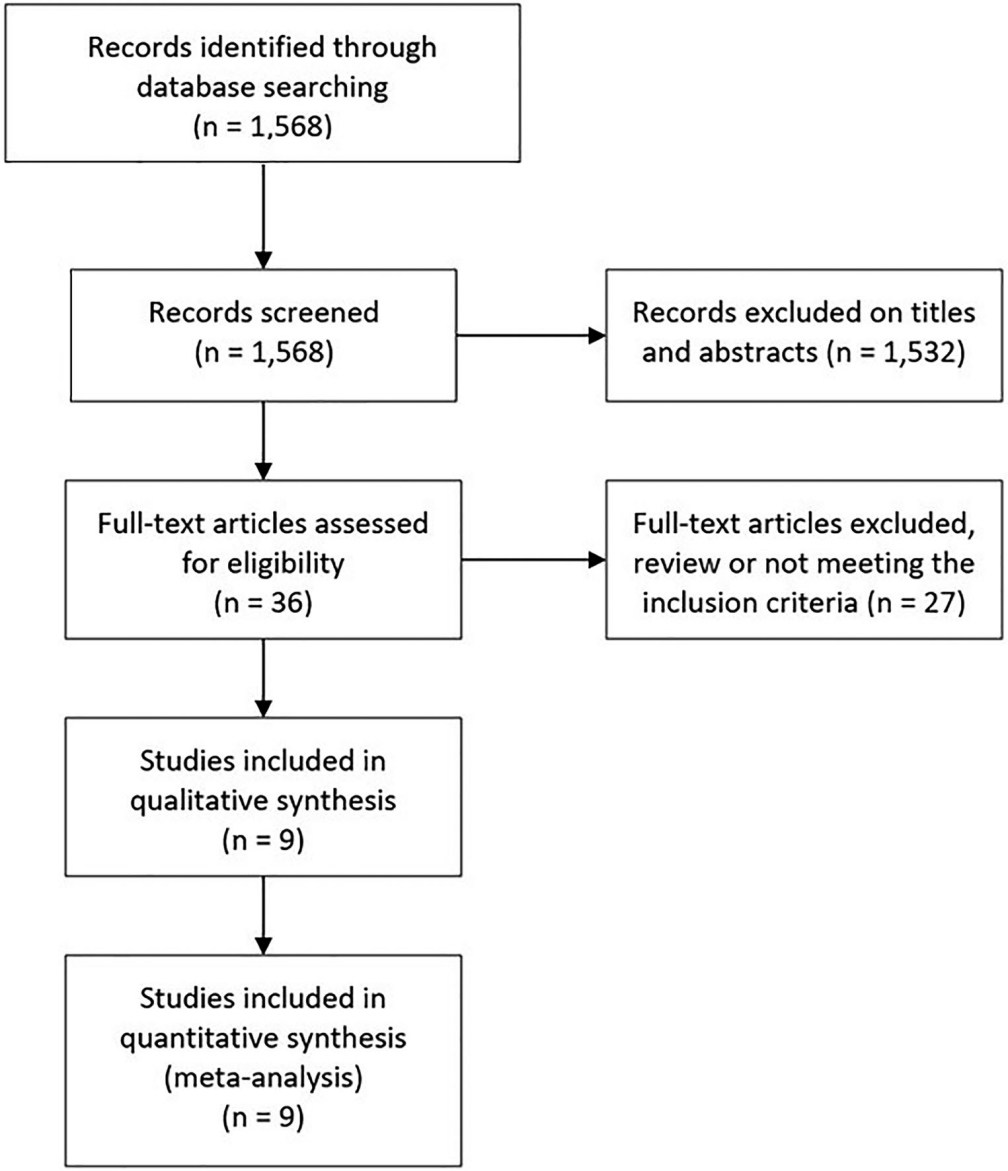
The flow-diagram showing the selection process of eligible studies for meta-analysis.

The initial search retrieved 1,568 studies. Of which, 91 were duplicates and 1,441 studies were excluded based on the titles, and abstracts screening, leaving 36 potential articles. After critical evaluation, 27 studies were further excluded, of which 13 were excluded because the review type; the other 14 studies were excluded because some the patients received percutaneous endoscopic interlaminar discectomy (PEID) or full-endoscopic interlaminar approach discectomy, which may introduce potential bias to our study. Finally, a total of nine studies were included, of which, eight randomized controlled trials (RCTs) and one retrospective study involving 1,679 patients with LDH (755 patients for PTED, and 924 patients for OLD) were included (Figure 1). The characteristics of the included studies were shown in Table 1.

**Table 1:** Characteristics of including studies.

Studies	Publication year	Study design	Procedures	Sample size	Age (years)	Gender (male/female)	Outcomes	Jadad Scores
Gadjradj PS et al. (20)	2022	RCT	PTED	179	45.3 ± 12.4	99/55	(1)(3)(4)(7)	7
			OLD	309	45.7 ± 11.3	180/58		
Zhang et al. (21)	2019	RCT	PTED	94	74.46 ± 6.01	46/48	(2)(3)(6)(7)	7
			OLD	94	73.02 ± 5.23	44/50		
Pan et al. (22)	2016	RCT	PTED	48	39.5 (22–58)	26/22	(1)(2)(3)(6)(7)	6
			OLD	58	42.8 (27–61)	31/27		
Xu et al. (23)	2017	RS	PTED	58	38.16 ± 5.93	34/24	(5)(7)	6
			OLD	87	36.75 ± 5.48	48/39		
Mayer et al. (14)	1993	RCT	PTED	20	41	12/8	(1)(4)(5)(7)	7
			OLD	20	41	14/6		
Hermantin et al. (13)	2018	RCT	PTED	30	39 (15–66)	22/8	(4)(5)(7)	6
			OLD	30	40 (18–67)	17/13		
GibsonR et al. (15)	2019	RCT	PTED	70	42 ± 9	30/40	(4)(7)	7
			OLD	70	39 ± 9	40/30		
Tao et al. (23)	2018	RCT	PTED	231	45.5 ± 4.8	126/105	(1)(2)(5)(6)(7)	6
			OLD	231	44.8 ± 4.6	133/98		
Tacconi et al. (24)	2016	RCT	PTED	25	43 (25–64)	13/12	(1)(4)(7)	7
			OLD	25	45 (21–69)	12/13		

PTED, percutaneous transforaminal endoscopic discectomy; OLD, open lumbar discectomy; RCT, randomized controlled trial.

Outcomes: (1) complication rates; (2) length of incision; (3) the amount of intraoperative blood loss; (4) length of operation; (5) excellent rates; (6) reoperation rates; (7) length of hospital stay.

### Primary outcomes

**Figure 2:**
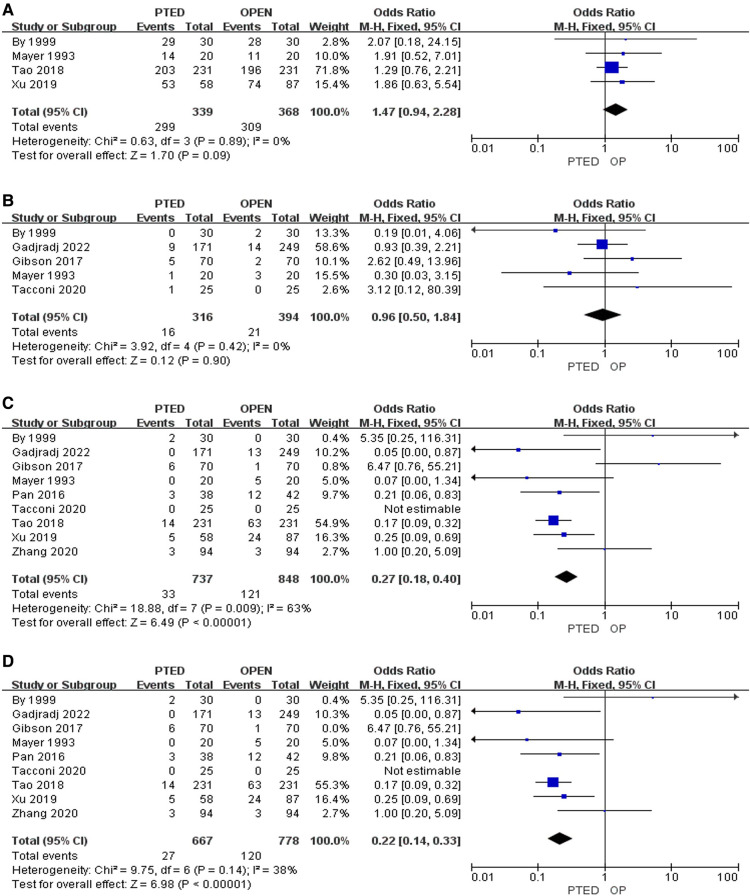
Pooling results of the PTED group and the OLD group. (**A**) Excellent rates; (**B**) Reoperation rates; (**C**) Complication rates; (**D**) Complication rates after sensitivity analysis.

Four studies reported excellent rates, which did not differ between PTED and OLD approaches (odds ratio [OR] = 1.47, 95% confidence intervals [CI]: 0.94–2.28, *P* = 0.09) (Figure 2A). Two studies reported on reoperation rates and did not find a difference between PTED and OLD groups (OR = 0.96, 95% CI: 0.50–1.84, *P* = 0.90) (Figure 2B). Notably, nine studies indicated that patients underwent PTED acquired less complications than those with OLD (OR = 0.27, 95% CI: 0.18–0.40, *P* < 0.001) but there was high heterogeneity (*I*^2^ = 68%, *P* = 0.009) (Figure 2C). After sensitivity analysis, the pooled OR of complication rates was 0.22 (95% CI: 0.14–0.33, *P* < 0.001) but without significant heterogeneity (*I*^2^ = 38%, *P* = 0.14) (Figure 2D).

### Secondary outcomes

**Figure 3:**
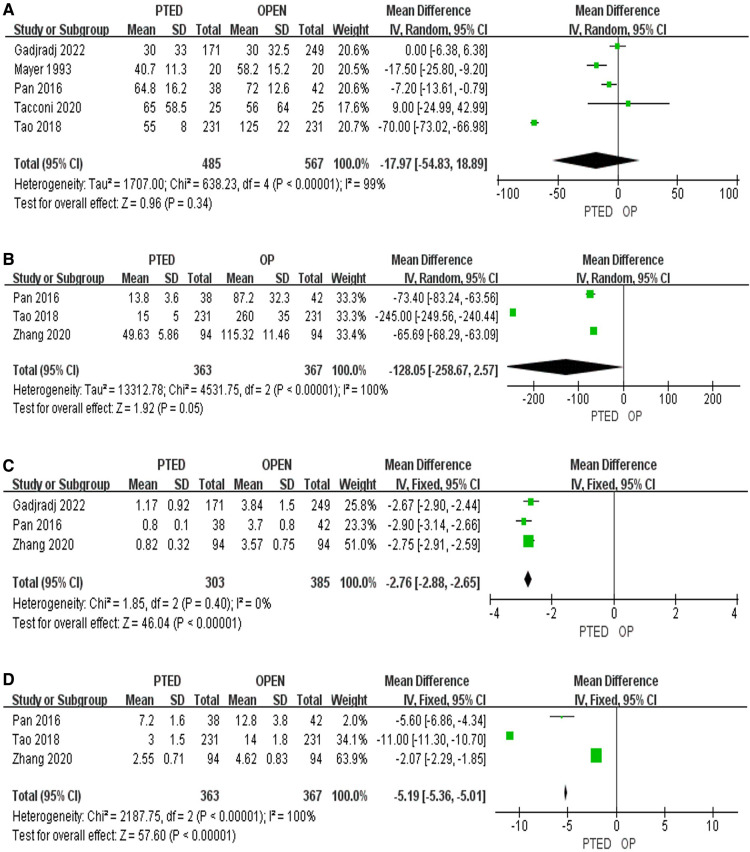
Pooling results of the PTED group and the OLD group. (**A**) Length of operation; (**B**) Amount of intraoperative blood loss; (**C**) Length of incision; (**D**) Length of hospital stay.

Length of operation [standardized mean differences (SMD) = −17.97, 95%CI: −54.83–18.89, *P* = 0.34], and the amount of intraoperative blood loss (SMD = −128.05, 95%CI: −258.67–2.57, *P* = 0.05) among patients who underwent PTED and OLD were insignificant, respectively (Figures 3A,B). Three RCTs reported the differences in length of incision, and patients who underwent PTED had smaller incision than patients who underwent OLD (SMD = −2.76, 95%CI: −2.88–−2.65, *P* < 0.001, Figure 3C). Same three RCTs also indicated that patients who underwent PTED had shorter hospital stay than patients who underwent OLD (SMD = −5.19, 95%CI: −5.36–−5.01, *P* < 0.001. Figure 3D).

### Publication bias

**Figure 4:**
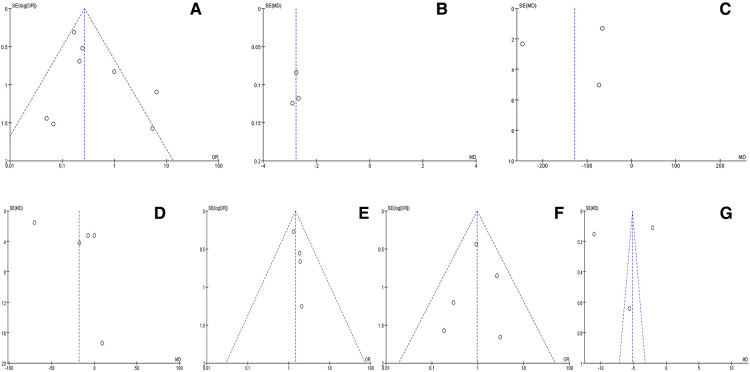
Funnel charts for publication bias. (**A**) Complication rates; (**B**) Length of incision; (**C**) Amount of intraoperative blood loss; (**D**) Length of operation; (**E**) Excellent rates; (**F**) Reoperation rates; (**G**) Length of hospital stay.

Funnel charts showed that there was no significant publication bias in all analysis (Figures 4A–G).

## Discussion

Our comprehensive systematic review which evaluated the effect of PTED vs. OLD for the treatment of LDH indicated that there is high quality evidence among included studies, and further meta-analysis suggests that complications were less frequently occurred in patients who underwent PTED. Furthermore, length of incision and length of hospital stay in PTED groups were significantly different from OLD groups. This may be because PTED approach does not require stripping the paravertebral muscles, preserves the original bone anatomy, and can perform the operation under local anesthesia, so it can be faster and more accurate than OLD approach. PTED can easily reach the responsible segment, and induce less trauma to the tissue so as to provide advantages for early postoperative recovery and discharge. Moreover, no superiority was found between PTED and OLD procedures with regard to excellent rates, reoperation rates, length of operation, and the amount of intraoperative blood loss.

Previously, other systematic reviews with different inclusion criteria and outcomes have been published (25–28). The current systematic review differs in that we only compared the studies which compared the effects of PTED vs. OLD approach. Study by Gadjradj et al., mainly focused on the comparison of pain scores and postoperative functional scores between PTED and traditional surgery for LDH (29), while study by Li et al., mainly focused on comparing the outcomes between foraminoscopic and no intervertebral discectomy (30). Our study is different from theirs, which systematically and comprehensively compared the PTED and OLD from seven postoperative indicators between PTED and OLD approach for LDH, including excellent rates, complication rates, reoperation rates, length of incision, length of operation, length of hospital stay, and the amount of intraoperative blood loss. Differences in outcomes between endoscopic discectomy and conventional surgery for lumbar disc herniation. Furthermore, the central-located high-canal compromised and high-grade migration herniations indicated a high rate of incomplete decompression treated with percutaneous interlaminar endoscopic discectomy (PIED) (31) for LDH. PIED has larger scope of the manipulation of the working cannula compared with PTED. Herein, it is better to use PIED in the treatment of migratory herniations. Further evidence based study on the effectiveness and safety of PIED in treatment of LDH should be performed.

Interestingly, there were only significant heterogeneity in meta-analysis on complication rates. This may be due to methodology such as study design, inclusion criteria, ethnicities and clinical characteristics such as lumbar intervertebral disc herniation and skill levels of surgical operators. In current study, after sensitivity analysis, there was no significant heterogeneity in field of pooled complication rates among including studies.

Present comprehensive systematic review and meta-analysis provided high quality evidence on the comparison of two procedure (PTED vs. OLD) for the treatment of LDH. However, there were some limitations in current study. First, although we performed that at least three studies are included in the comparison for each indicator, the number of trials involved in some comparisons are relatively small. Second, subgroup analysis disparity on the inclusion criteria, the baseline characteristics of patients and the follow-up period in different trials were not conducted due to insufficient data. Third, none of the included studies reported postoperative survival rate, and mortality, thus, long-term efficacy and safety evaluation could not be performed. In view of this, the effect of PTED procedure in the treatment of LDH still needs to be confirmed by long term, large sample size, high quality, rigorous study design, and future research should strictly follow the CONSORT-2010 standard to report relevant information. Furthermore, we only evaluated the publication bias, but without risk of bias analysis due to the limited including studies. Last but not least, although there are some comparative studies between minimally invasive endoscopic surgery and open surgery in the treatment of LDH. Importantly, some of the advantages of minimally invasive endoscopic surgery, including less paravertebral muscle injury, rapid recovery, shorter hospital stay, and less blood loss, are well established, it makes more sense to do a transforaminal approach and a translaminar approach for lumbar discectomy in future.

## Conclusion

PTED can be considered sufficient to achieve good clinical outcomes on the complication rates, length of incision, and length of hospital stay compared with OLD. Both PTED and OLD are able to achieve similar outcomes on excellent rates, reoperation rates, length of operation, and the amount of intraoperative blood loss for patients with LDH. For patients who meet surgical indications, we recommend the use of PTED approach for the treatment of LDH.

## Data Availability

The raw data supporting the conclusions of this article will be made available by the authors, without undue reservation.
